# An evaluation of GO annotation retrieval for BioCreAtIvE and GOA

**DOI:** 10.1186/1471-2105-6-S1-S17

**Published:** 2005-05-24

**Authors:** Evelyn B Camon, Daniel G Barrell, Emily C Dimmer, Vivian Lee, Michele Magrane, John Maslen, David Binns, Rolf Apweiler

**Affiliations:** 1 European Molecular Biology Laboratory-European Bionformatics Institute (EMBL-EBI), Wellcome Trust Genome Campus, Hinxton, Cambridge, CB10 1SD, UK

## Abstract

**Background:**

The Gene Ontology Annotation (GOA) database  aims to provide high-quality supplementary GO annotation to proteins in the UniProt Knowledgebase. Like many other biological databases, GOA gathers much of its content from the careful manual curation of literature. However, as both the volume of literature and of proteins requiring characterization increases, the manual processing capability can become overloaded.

Consequently, semi-automated aids are often employed to expedite the curation process. Traditionally, electronic techniques in GOA depend largely on exploiting the knowledge in existing resources such as InterPro. However, in recent years, text mining has been hailed as a potentially useful tool to aid the curation process.

To encourage the development of such tools, the GOA team at EBI agreed to take part in the functional annotation task of the BioCreAtIvE (Critical Assessment of Information Extraction systems in Biology) challenge.

BioCreAtIvE task 2 was an experiment to test if automatically derived classification using information retrieval and extraction could assist expert biologists in the annotation of the GO vocabulary to the proteins in the UniProt Knowledgebase.

GOA provided the training corpus of over 9000 manual GO annotations extracted from the literature. For the test set, we provided a corpus of 200 new *Journal of Biological Chemistry *articles used to annotate 286 human proteins with GO terms. A team of experts manually evaluated the results of 9 participating groups, each of which provided highlighted sentences to support their GO and protein annotation predictions. Here, we give a biological perspective on the evaluation, explain how we annotate GO using literature and offer some suggestions to improve the precision of future text-retrieval and extraction techniques. Finally, we provide the results of the first inter-annotator agreement study for manual GO curation, as well as an assessment of our current electronic GO annotation strategies.

**Results:**

The GOA database currently extracts GO annotation from the literature with 91 to 100% precision, and at least 72% recall. This creates a particularly high threshold for text mining systems which in BioCreAtIvE task 2 (GO annotation extraction and retrieval) initial results precisely predicted GO terms only 10 to 20% of the time.

**Conclusion:**

Improvements in the performance and accuracy of text mining for GO terms should be expected in the next BioCreAtIvE challenge. In the meantime the manual and electronic GO annotation strategies already employed by GOA will provide high quality annotations.

## Background

The number of proteins requiring functional characterization in the UniProt Knowledgebase [1] is still growing. Although proteins can be electronically annotated using existing resources [2], the most reliable and detailed annotation is still manually extracted from the literature by a team of experts. The problem with knowledge archived in the literature is that it is represented in scientific natural language where a variety of text phrases can be used to describe the same concept. Traditionally, this information could be deciphered by humans but was not easy to interpret computationally. Furthermore, the number of biological databases has also increased so that up-to-date annotation relies on the ability to integrate information from multiple sources.

Currently, one of the most important advances in database annotation, querying and interoperability is the development and use of structured vocabularies. In this regard, one of the most successful is the 'Gene Ontology' (GO) [3,4]. Since 2001, the GOA database [2,5] at the EBI has used GO to provide consistent descriptors for proteins in its UniProt Knowledgebase in the categories of *molecular function*, *biological process *and *cellular component*.

With the success of GO's integration into the analyses of microarray [6,7] and mass spectrometry data [8], academic and pharmaceutical institutions are keen to fast-track the assignment of GO terms to large datasets. Consequently, a new generation of tools have been developed which aim to predict GO annotations using interacting networks [9], existing protein features [2,10], sequence [11] and semantic similarities [12]. Numerous text mining systems [13-15] have also attempted this task or reported results on aspects of this task.

While some of these tools are useful, others demonstrate a lack of understanding of how GO is used and queried by a biologist. For example, the GO term 'cell adhesion' (GO:0007155) has been experimentally verified as a process involving the protein ICAM1 but to assign that GO term automatically to every paper that mentions the protein ICAM1 is simply incorrect. Every article that mentions ICAM1 will not experimentally verify that process within its text; instead, it might simply describe the sequence. Annotating GO terms to biomedical literature in this way is not useful to curators, as the GO term is often not attached to a 'relevant' paper. For developers of automatic information extraction and retrieval techniques, however, this strategy might form part of a useful intermediate step to limit the number of GO terms to be searched in a given piece of text.

So what do GO curators really need? A useful tool would allow curators to retrieve all 'relevant' papers which report on the distinct features of a given protein and species and then to locate within the text the experimental evidence to support a GO term assignment. Given that GO is not designed for text mining, it is of no surprise that exact text strings of many of the 18,000 GO terms will not be found verbatim in the literature. Despite these difficulties, GOA is often asked to evaluate various automatic GO retrieval and extraction systems. To encourage their comparison and development and to save time in individually evaluating the different strategies, the GOA team was delighted to take part in task 2 of the BioCreAtIvE (Critical Assessment of Information Extraction systems in Biology) challenge.

BioCreAtIvE task 2 was designed to assess if automatically derived classification using information retrieval and extraction could assist biologists in the annotation of the GO terminology to proteins in UniProt. For the training set, participants were provided with papers linked to GO annotations from human proteins already publicly available [5]. For the test set, we annotated 286 blind (not yet released) human proteins with GO terms using the full text of 202 *Journal of Biological Chemistry *articles. We manually evaluated 22,000 segments of text, which were provided to support the correct GO term and protein predictions. In this paper, we give a biological perspective on the evaluation, explain how we manually annotate GO using literature and offer some suggestions to improve the precision of future text retrieval and extraction techniques. Finally, we provide the results of the first inter-annotator agreement study for manual GO curation, as well as results assessing our current electronic GO annotation strategies, to help to establish a threshold for the text mining technology.

## Methods, Results and Discussion

### Current electronic and manual GO annotation strategies at the EBI

One of the distinguishing features of the UniProt Knowledgebase is the high level of annotation and database cross-references that are integrated with each entry. It therefore makes sense that the large-scale assignment of GO terms to the proteins in UniProt should exploit the existing knowledge stored in these entries [2]. Enzyme Commission (EC) numbers and Swiss-Prot keywords have been manually curated into UniProt entries for many years. A manual mapping of GO terms to these existing vocabularies allows GO terms to be retrofitted to appropriate UniProt records. Similarly, UniProt records contain cross-references to the InterPro and HAMAP (High-quality Automated and Manual Annotation of microbial Proteomes) databases [16,17]. This is because the associated sequence contains features (signatures and domains) which provide evidence for their membership in a particular protein family. Based on a review of the literature of the well-annotated family members, GO terms are manually mapped to InterPro and HAMAP records. These mappings are released monthly with every GOA release and provide a useful first pass electronic GO annotation in the GOA database. As of March 2005, this strategy provided 69% GO coverage of UniProt records for over 85,000 species [5]. Surprisingly, BioCreAtIvE participants did not appear to exploit these released GO annotations to help limit the GO lineages that might be found in the test set papers. Later in this paper, we will explore the accuracy of these electronic GO annotations and compare the results to the text mining systems used in BioCreAtIvE.

Electronic techniques are efficient in associating high-level GO terms to large datasets. On the other hand, manual curation provides more reliable and detailed GO annotation but is slower and more labour-intensive. It is clear that the manual curation process requires automatic assistance. However, before attempting to develop strategies to help curators make more rapid GO assignments, it is important to first understand current manual approaches.

Each GO consortium member uses slightly different techniques in locating papers suitable for manual GO annotation [18,19]. The following describes the approach of the GOA curators. First we have to decide which human proteins to prioritize for GO annotation. We concentrate on 3 categories, (a) those which have no GO annotation, (b) those which have disease relevance and (c) those which are important for microarray analyses. Having chosen the protein accession to annotate, we now need to find relevant scientific papers. The first step is to decide if the papers already linked within the UniProt entry are relevant for GO annotation. The decision on whether to read the full text of a paper is based on the curator's interpretation of the text used in the paper title or abstract. The journals cited in UniProt/TrEMBL records are inherited from EMBL/DDBJ/GenBank databases [20] and so may describe the sequence rather than GO function, process or component. Papers that reference the sequence are accompanied by a remark located in the reference position (RP) line, which says 'SEQUENCE FROM N.A.' (nucleic acid). On the other hand, UniProt/Swiss-Prot records are manually supplemented with documents to support the annotation stored in the comment (CC) lines. In these cases, the remark in the RP line might also indicate the type of information extracted from a paper e.g. 'SUBCELLULAR LOCATION', 'FUNCTION', 'INTERACTION'. It should be noted, however, that the use of the word FUNCTION in Swiss-Prot is not the same as 'Molecular Function' usage in GO. Frequently, GO process terms can be extracted from FUNCTION CC lines.

In addition to the papers archived in the UniProt records, the NCBI PubMed advanced search [21] is queried to find papers that support supplementary GO annotation. Various combinations of the gene and protein, full and abbreviated names are searched. Initially, searches are limited to 'Title' or 'Title/abstract' and to 'Human entries only'. Electronic GO annotation and information in UniProt/Swiss-Prot CC lines often provide curators with an insight into the types of functions that could be extracted from the literature. With this information to hand, curators are able to refine their search options to find more than enough relevant papers for GO annotation. In GOA, our current aim is to find the most recent papers which provide experimental evidence for the unique features of a given protein. Our approach is protein-centric rather than paper-centric as it is not necessary to read all the relevant papers that might be used to assign the same GO term. In the future, however, adding more papers to experimentally verify a given function will provide greater confidence to the GO annotations. A good source of a complete set of functional annotations is often retrieved from recent review articles. These reports often have links to relevant papers with experimental verification. Any papers that report new data are fed back to the UniProt curators to add to the original entry.

Most GO Consortium members would agree that the most difficult task in searching the literature is finding papers that have experimental information for a given species. Often, the species 'name' (e.g. human) is not mentioned in the 'Title' or 'Abstract' and occasionally, not directly mentioned in the full text. On these occasions, the method section of the paper has to be read and perhaps the taxonomic origin of a cell line identified before any attempt at GO curation. Filtering 'Human entries only' via PubMed is not always accurate. In addition, authors do not always cite the most up-to-date gene nomenclature e.g. use of upper case letters for human gene symbols [22]. This is likely to affect the precision of automatic 'gene product' entity extraction techniques.

### Finding functional annotation and choosing the correct GO term

Once a relevant paper is found, the full text is read to identify the unique features of a given protein. The majority of papers will mention more than one protein; however, a curator will concentrate on capturing the information pertinent to the main protein chosen for annotation. Most curators still prefer to print out papers rather than view papers online. This is simply to limit computer eye strain and because a curator can quickly scan and select the most relevant parts of the document for curation. Words or short phrases which can be converted to GO terms are highlighted by hand and the correct GO term identifier (ID) is documented in the paper margins for review.

GO terms are chosen by querying the GO files with the QuickGO web browser [2,23] or with a local copy of DAG-Edit (official GO editor with browsing capability)[3]. Before assigning a GO term, the definition must be read to check its suitability. Obsolete GO terms (children of *obsolete molecular function *(GO:0008369), *obsolete cellular component *(GO:0008370), *obsolete biological process *(GO:0008370)) are not used in annotation. When electronic or manual GO annotations become obsolete, they are manually replaced with an appropriate term [24]. The reason for the obsoletion and suggestions for replacement GO terms are documented in GO comment lines. If a useful term is missing from the ontology, an existing GO term is in the incorrect hierarchical position or a definition needs to be refined, a curator request is sent to the GO editorial office using SourceForge [3,25].

The GO Consortium avoid using species-specific definitions for GO nodes; however some function, processes and component are not common to all organisms. Inappropriate species-specific GO terms (e.g. germination GO:0009844) should not be manually annotated to mammalian proteins. Sometimes these inappropriate terms can be distinguished by the *sensu *(in the sense of) designation (e.g. embryonic development (sensu Magnoliophyta, GO:0009793). Curators are cautious when manually assigning these terms. To avoid generating inappropriate GO term assignments, the text mining community should read the GO Consortium documentation on the subject [26].

If a curator is unsure of which process term should accompany a function term, they can consult the 'Often annotated with' section of the QuickGO browser. Here, GO terms that are assigned in tandem are displayed. These are also referred to as common concurrent assignments and are calculated on our existing manual and electronic GO annotations [2].

It is important to note that GO terms are often extracted from particular regions of a paper. Furthermore, according to GO Consortium rules, each GO annotation must be accompanied by a PubMed identifier and one of 10 manual GO evidence codes [27]. Table 1 shows the important regions of the paper for GO annotation. The 'Materials and Methods' section of a paper is only used to identify the species of protein used in the research and to determine which GO evidence code should be used. It is not used to extract functional annotation. Furthermore, curators often piece together information from different parts of a document to reinforce a decision to annotate. GO annotation is not associated with UniProt entries until the entire article is read.

If no functional annotation can be found for a given protein after an exhaustive literature search, the GO terms *molecular_function unknown *(GO:0005554), *biological_process unknown *(GO:0000004) or *cellular_component unknown *(GO:0008372) can be assigned with GO evidence code ND ('No Data').

### BioCreAtIvE task 2 training set

It is clear from the above that the manual GO annotation effort has many steps, which could be assisted by automatic information extraction techniques. For these reasons, BioCreAtIvE organizers designed a biologically motivated task which asked systems to identify the proteins in the text, to check if any functional annotation was present and to return the GO term ID representing this information and the evidence text that supported the annotation.

To train systems to perform this task accurately, thousands of manual GO annotation examples were required. The training data provided to participants is documented online [28]. Essentially, the training set was extracted from the publicly available non-redundant human GO annotation dataset (gene_association.goa_human.gz) [5]. It consisted of approximately 9000 manual GO associations linked to UniProt accessions, PubMed IDs and GO evidence codes. It was advised that GO annotations with GO evidence codes '*Inferred from Sequence/Structural Similarity*' (ISS), '*Inferred by Curator*' (IC) judgment and '*No Data*' (ND) should be ignored.

It is important to note that historically, most of the human GO annotations in the GOA database were generated before 2002. Approximately 6000 manual annotations were integrated from the former Proteome Inc. (now Incyte Genomics), which may or may not have been extracted from full text, while an additional 3000 proteins were annotated by UniProt curators from abstracts only, as part of a fast-tracking strategy. These annotations can be identified in the GOA database with GO evidence codes NAS or TAS [27]. Since 2002, full text articles are always read but the annotation and thus the creation of a large and useful training set is slow. These data can be identified in GOA by extracting terms with the GO evidence code, IDA, IEP, IMP, IGI or IPI [27]. As a result, the number of useful training data will be relatively small and will represent relatively few GO terms. Furthermore, the relevant passages of the text used in curation were not marked in the training set. As such, the training data was not equivalent to the task allocated (marked passages not provided). This was unavoidable given current annotation approaches and may have affected the precision and recall abilities of some systems in the first BioCreAtIvE challenge. However a positive outcome of the BioCreAtIvE evaluation is that marked passages useful in GO curation have been manually verified and made available for future training.

### BioCreAtIvE task 2 test set

To create the BioCreAtIvE test set, GOA was asked to associate 200 papers with human proteins and GO terms. The *Journal of Biological Chemistry*' (JBC) (dated between years 1998–2002) was chosen by the organizers because of an arrangement to use the full text openly and freely. We chose a set of JBC articles already associated with human proteins within the UniProt flat files. This set was then filtered for proteins that had no previous manual GO annotation. These criteria ensured that the annotations created for the test set would be new to both the GOA database and the participants. In total, a list of 286 UniProt accessions together with the PubMed ID of the article was distributed to 3 curators. A new GO annotation tool was created to collect the GO associations and to ensure that they would not be released or touched by other UniProt curators not involved in the BioCreAtIvE challenge. The test set took the curators one month to complete (approx. 10–15 papers per day). During this period, 923 distinct GO terms were extracted from text within the papers. The evidence text was highlighted on paper and therefore not in a format for machine processing. On average, each protein had 9 GO annotations. During the curation process, these GO annotations were associated with the proteins from 37 other mammalian species (e.g. mouse, pig, dog, rat, horse) based on their sequence similarity to the human proteins. To prevent participants from back-extrapolating the test set annotations, associations with the evidence code 'ISS' were also suppressed from GOA releases. In Table 2 an example is provided which shows that the human protein for 'Estrogen receptor beta'(ESR2_HUMAN, Q92731) has been annotated with the GO term 'estrogen receptor activity' using PubMed ID: 11181953. Because this protein has high-level sequence identity with the mouse ortholog, the original GO annotation has been transferred from the human entry to the mouse 'Estrogen receptor beta' protein (ESR2_MOUSE, O08537, Table 2) with the GO evidence code 'ISS'. The 'with' column indicates the source (ESR2_HUMAN, Table 2) of the GO annotation. It would be easy to extrapolate back the original GO annotation that was assigned to the human protein.

One difficulty in creating the test set was that curators were often restricted to a single article per protein. Normally, a curator would seek verification of author statements from more than one paper. As a result, some articles were slightly over annotated compared to the normal curation process.

The test set was released to the BioCreAtIvE organizers on 3 November 2003. It was advised that participants should not use versions of GO archived in the CVS repository beyond this date. This was to ensure that the same GO ontology files were available to both the annotators and participants. The test set was suppressed from the monthly GOA release until January 2004.

### Evaluation tool and criteria

BioCreAtIvE organizers created an online evaluation tool for task 2. For subtask 2.1, the tool displayed the UniProt accession in the test set, along with associated 'known' GO terms and documents. Participants were expected to return a segment of text (the evidence text) from the document that supported the annotation of the 'known' GO term. The provision of evidence text was critical for the evaluators as it provided a basis for rejecting or accepting that finding. Evidence text was visible to evaluators by means of a red text highlight. The full text surrounding the evidence text was also visible in black or blue font. The evaluation tool was easy to use and was designed with the evaluators to closely resemble a curation aid that might develop from this technology. Two GOA curators evaluated subtask 2.1. There were 9 distinct users for this task but 21 separate runs were submitted for evaluation.

**Figure 1:**
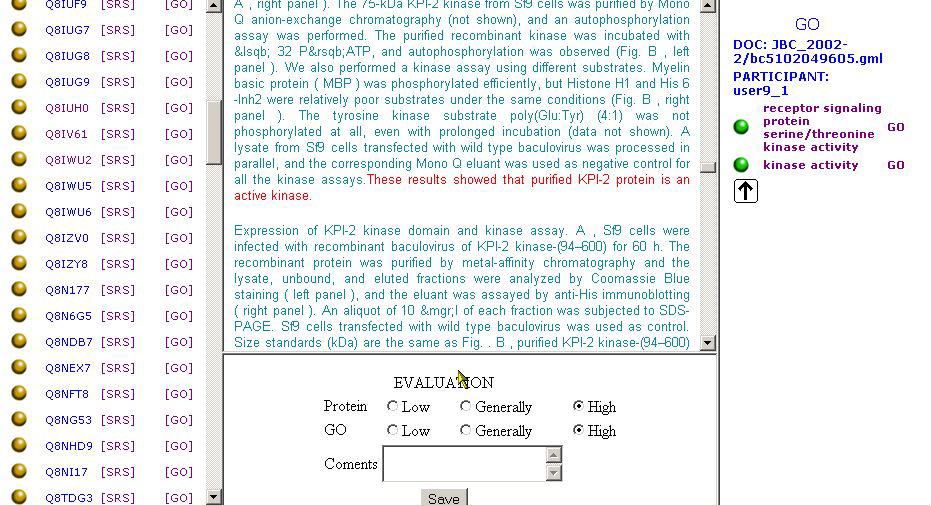
**BioCreAtIvE Evaluation Tool (subtask 2.2). **showing GO annotation of 'kinase activity' GO:0016301 (right tool bar) by user 9-1 with supporting text evidence (central panel). The left tool bar shows the UniProt accession number, in this case Q8IWU2 has been annotated. Q8IWU2 represents a KPI-2 protein so the user has been evaluated based on the evidence text as 'high' for GO term prediction and 'high' for representing the correct gene product. The user also uses this sentence to predict the GO term 'receptor signaling protein serine/threonine kinase activity'(GO:0004702). Although that GO annotation is correct for this protein the evidence text supplied does not support that level of detail. The same evidence text was evaluated as 'general' for the GO term prediction of GO:0004702 (same lineage as correct GO term 'kinase activity') and 'high' for representing the correct gene product.

In the second subtask (2.2), participants were given the document and the associated UniProt accession and asked to return evidence text to support their system's GO predictions for that protein (Figure 1). Participants were aware of how many GO process, function and component terms the curator had manually extracted from the document. This task was understandably more difficult for the participants and was evaluated by a single curator. There were 7 participants for this subtask but 18 separate runs were submitted for evaluation. In total, 30,000 individual results were submitted for review. Because of the lack of time and the expense of a 2 month evaluation, only 22,000 text highlights were assessed. In these cases, entire proteins were skipped so that all participants were affected in the same way. In both subtasks, numbers 1–20 anonymously represented the participants. There was a problem visualizing the text highlights in the tool for some users. Without the supporting text, GO and protein predictions could not be evaluated. Towards the end of the evaluation, some of the highlighting problems were fixed and 40 proteins with GO associations were re-evaluated for user numbers 17 and 7.

The curators made two separate evaluations of the evidence text: Did it support the correct GO term? Did it support the correct protein association? To ensure the consistency of evaluations, criteria were agreed amongst the 3 evaluators and BioCreAtIvE organizers (see Table 3).

It was clear from the evaluation and BioCreAtIvE workshop (March 2004) that not all participants understood the content of GO or how it is used during annotation. The common mistakes collected by curators are presented in Table 4 together with some suggestions for improvements. We hope this will be helpful in future BioCreAtIvE challenges. The major problem that slowed down the evaluation considerably was that that the vast majority of text highlights were too long. Even though the precision in GO predictions was usually low, the evaluators had to re-read the passages for each GO term, participant and run. These results, however, reflect the difficulty systems had in finding a piece of text that contained reference to both the query protein and the functional annotation. It may be more useful to curators and future evaluations if these entities are highlighted separately within the full text.

### Inter-annotator agreement

After the BioCreAtIvE evaluation, GOA was asked to perform an inter-annotator evaluation to measure how consistently the curators could precisely recall GO annotation from the literature. This was important to judge the ceiling on performance that can be expected from the text mining systems for the same task. To speed up the study, each of the 3 evaluators randomly chose 10 papers that they had already curated during the creation of the BioCreAtIvE test set and passed them to another curator. The second curator extracted GO terms from the text blindly. In total, 30 papers were co-curated. The 2 sets of GO terms extracted from the text were divided into 3 categories (a) exact term match (GO term was exact match to that chosen by the second curator), (b) same lineage (GO term was parent or child to that chosen by the second curator), (c) new/different lineage (GO term was *not *a parent or child to that chosen by the second curator). At the end of the study, the 3 curators evaluated together the GO terms extracted in the 3 categories. Results indicate that there is 39% chance of curators exactly interpreting the text and selecting the same GO term, a 43% chance that they will extract a term from new/different lineage, and a 19% chance that they will annotate a term from the same GO lineage (Table 5). See additional file 1 for the original data used to perform this analysis. This variation is not surprising since curators are taught to annotate according to their individual level of confidence. This will vary according to how well the topic covered by the article matches the curators' biological background. It was also clear that 3 curators together would create a more accurate and complete GO annotation of a protein than an individual. This phenomenon was light-heartedly referred to as the 'superhuman complex' at the BioCreAtIvE Workshop. Variation is acceptable between curators but inaccuracy is not. Fortunately, the inter-annotator agreement showed that 94% of the time curators were precisely extracting GO annotation from the literature. 72% of the time curators recalled all possible valid GO terms from the text. This creates a particularly high threshold for text mining systems which, in task 2.2, precisely predicted GO terms only 10–20% of the time.

### Evaluation of in-house electronic GO annotation techniques with manual annotations created for the task 2 test set

As described earlier, the large-scale GO annotation of UniProt entries involves electronic techniques based on transitive mappings (InterPro2GO, SPKW2GO and EC2GO). It was of interest to GOA to also evaluate the precision of these annotation strategies. Taking the manual GO annotation created for the BioCreAtIvE test set, we again compared the number of times the different electronic techniques predicted GO terms exactly, with the same lineage and less granularity (parent of manual GO annotation), same lineage and greater granularity (child of manual GO annotation) or new lineage. It should be noted that electronic predictions that exactly matched or represented a parent term of a manually annotated term were assumed to be correct. Electronic GO predictions that represented a new lineage or a child term to those chosen manually could be potentially correct or incorrect. This is because the GO annotations represented in the BioCreAtIvE test set were based on the curation of just a single article and therefore not fully curated. In agreement with GOA release statistics, InterPro2GO (635 annotations) provided the most GO coverage of the test set followed by SPKW2GO (385 annotations) and EC2GO (27 annotations), data not shown. Because the GO function terms predicted by the EC2GO mappings were quite deep/final node GO terms, it was not surprising that 67% of the time they exactly matched the manual GO annotation (Table 6). The InterPro2GO (43%) and SPKW2GO (44%) mappings, however, were more likely to predict a higher level/ less granular term than those chosen manually. Given that this was an automatic evaluation, the precision of electronic GO term predictions was calculated based on new or more granular GO terms being either correct or incorrect. As a result, a precision range is presented for each electronic strategy. In the worst case scenario, InterPro2GO, SPKW2GO and EC2GO precisely predict the correct GO term 60 to 70% of the time. On the other hand, all strategies were capable of up to 100% precision. The reason for this level of accuracy is because these electronic strategies rely on a manual mapping step based on quite high level GO terms. As stated earlier, it was noticed by curators that the BioCreAtIvE systems evaluated tended to over-predict GO terms. It is more important for database curation to be accurate than to have complete coverage.

To further evaluate how precise our electronic strategies were, we manually evaluated a random set of 44 proteins that had both electronic and manual GO annotation. This time, we verified whether the GO predictions were correct or incorrect. There was little difference in the precision of each strategy and our electronic annotation was between 91–100% precise (Table 7). These results suggest that, at the moment, our current large-scale GO annotation protocol is more accurate than text mining technologies presented during the first BioCreAtIvE challenge.

## Summary

The GOA database currently provides 69% GO coverage of the UniProt Knowledgebase using in-house electronic and manual annotations as well as annotations integrated from GO Consortium members including MGI [18], SGD [19] and FlyBase [29]. The analyses presented in this paper indicate that these techniques have high precision (90–100%) but every year, the number of new proteins requiring GO annotation increases. As such we need to develop new techniques to increase GO coverage without compromising on high quality annotation. We used the BioCreAtIvE functional annotation challenge as an opportunity to help the research community, which might in turn ultimately help us to speed up our curation progress. The results of BioCreAtIvE task 2 indicate that the prediction of GO terms and provision of supporting text evidence is a difficult task. However, given the simple mistakes that were made and the creation of relevant training data during the evaluation [30], the improved performance of text mining systems in the next BioCreAtIvE challenge is inevitable. To supplement this training data and limit the expense of future evaluations, a tool that would allow curators to highlight and even link several important sentences that support a GO annotation might be useful. Ultimately, future functional annotation challenges could be evaluated semi-automatically by matching the highlighted regions of text.

Although GO was not designed with text mining in mind, it does try to create a vocabulary for biological research that could be deciphered by both humans and machine processing. The complications in matching exact GO terms in the literature might be resolved when the GO Consortium implement their plans to decompose the GO phrases into individual words or concepts and properties and by the mapping of more synonyms to GO terms [31].

## Conclusion

Improvements in the performance and accuracy of text mining should be expected in the next BioCreAtIvE challenge. In the future we hope it will offer a useful supplement to the manual and electronic techniques already employed by GOA.

## Authors' contributions

RA heads the UniProt Knowledgebase and organized the collaboration with the BioLink group. EBC coordinates the manual curation of GOA database, drafted the manuscript and performed the statistical analysis (Table 1, 2, 3, 4, 5, 6). DGB coordinates the automatic classification of GO terms in the GOA database and worked closely with JM and DB to create a GO annotation tool used to build the BioCreAtIvE task 2 test set. DGB also generated the data for Table 5. EBC, ECD, and VL helped to create the training and task 2 test set as well as evaluating the task 2 subtasks and the reliability of in-house electronic techniques. MM coordinates the manual curation of UniProt at EBI and was involved in creating the training set and in the design of the BioCreAtIvE test set. All authors read and approved of the final manuscript.

## Supplementary Material

Additional File 1 This shows further details of the inter-annotator agreement. It contains individual counts for each UniProt accession and PubMed Identifier that was co-curated. Click here for file

## Figures and Tables

**Table 1:** Important regions of a paper for GO annotation and the type of GO evidence codes that can be typically extracted from these regions.

**Region of Paper**	**GO Evidence Code**
Title/Abstract	Non-traceable author statement (NAS) Traceable author statement (TAS)
Introduction	Non-traceable author statement (NAS) Traceable author statement (TAS)
Results	All GO evidence codes [27]
Discussion	All GO evidence codes [27]
Figure Legend	All GO evidence codes [27]
Materials and Methods	Identify species (via cell line). Identify GO evidence code according to experiment used.

**Table 2:** 

**ESR2_HUMAN (Q92731)**					
**GO TERM NAME**	**GO ID**	**SOURCE**	**EVIDENCE**	**REFERENCE**	**WITH**

estrogen receptor activity	GO:0030284	UniProt	TAS	PubMed: 11181953	

**ESR2_MOUSE (O08537)**					

**GO TERM NAME**	**GO ID**	**SOURCE**	**EVIDENCE**	**REFERENCE**	**WITH**

estrogen receptor activity	GO:0030284	UniProt	ISS	PubMed: 11181953	ESR2_HUMAN Q92731

**Table 3:** Evaluation criteria for GO and protein predictions.

**Evaluation**	**Criteria for GO term assignment**	**Criteria for protein association**
**High**	The GO term assignment was correct or close to what a curator would choose, given the evidence text.	The protein mentioned in the evidence text correctly represented the associated UniProt accession (correct species).
**General**	The GO term assignment was in the correct lineage, given the evidence text, but was too high level (parent of the correct GO term) e.g. *biological_process *or too specific.	The evidence text did not support annotation to the associated UniProt accession but was generally correct for the protein family or orthologs (non-human species).
**Low**	The evidence text did not support the GO term assignment. **Note: **The GO term may have been correct for the protein but the evidence text did not support it.	The evidence text did not mention the correct protein (e.g. for Rev7 protein (ligand) incorrect evidence text referred to 'Rev7 receptor') or protein family.

**Table 4:** Summary of mistakes and curator comments following the task 2 evaluation.

**Mistakes**	**Suggestion/Comment**
Predicting obsolete GO terms	Strip obsolete GO terms, i.e. children of *obsolete molecular function *(GO:0008369), *obsolete cellular component *(GO:0008370), *obsolete biological process *(GO:0008370) [25]
Predicting GO terms from Materials and Methods e.g. 'pH' value yielded 'pH domain binding' (GO:0042731), 'CHO cell line' yielded numerous GO terms containing 'acetyl**cho**line'.	Only look in certain sections of the paper for features. See Table 1 for GOA.
Predicting plant GO terms to human proteins e.g. *germination *(GO:0009844)	Look at GO Documentation on *sensu *[24] and strip out unnecessary GO terms.
Highlighting too much text	Set limit on evidence text highlight to be useful for curators. Limit to <5 lines.
Over-predicting GO terms from one line of text	More important to curator to choose a higher level term that is correct than to be too specific and incorrect.
Common GO terms predicted out of context e.g. text 'mapped to chromosome 3q26' yielded GO component term 'chromosome' GO:0005694. Text indicates chromosome number, not where the protein functions. e.g. text '249 amino acid' yielded multiple GO terms i.e. 'amino acid activation' GO:0043038.	Most papers will mention chromosome location and the amino acid length of a sequence. Do not predict GO terms from text if words 'chromosome' or 'amino acid' in evidence text is accompanied by a number.
Choosing first paragraph of paper as supporting text	Although a lot of information can be found in introduction of paper, the task was to choose the highlight which supported the GO term. Whole paragraph highlights do not speed up the curation process. Limit to <5 lines.
Difficulty in interpreting word order e.g. 'RNA binding protein' yielded the incorrect GO prediction 'protein binding'	
Difficulty in predicting correct taxonomic origin of protein.	This can also be difficult for a curator, given lack of evidence in text.
Too many low confidence runs	Only submit data with high confidence level for evaluation. Limit participants to their best run/technique. (little difference between runs, repeat evaluations)

**Table 5:** Inter-annotator agreement.

**GO terms**	**Curator 1 +2**	**Curator 1+3**	**Curator 2+ 3**	**Average**
**Exact**	47	35	35	39
**Same Lineage**	15	20	19	18
**New Lineage**	56	39	35	43
**Correct**	107	91	85	94
**Incorrect**	11	3	4	18
**TOTAL**	118	94	89	100
**Precision**	0.91	0.96	0.96	0.94
**Recall**	0.70	0.72	0.73	0.72
**F-measure**	0.79	0.82	0.83	0.82

Where precision is the fraction of manual GO term annotations that are correct (number of correct annotations / (number of correct annotations + number of incorrect annotations). Recall is defined as the fraction of correct GO term annotations that were successfully retrieved during manual annotation (number of correct annotations / number of correct annotations + (number of annotations from new lineage - number of incorrect annotations). New lineage annotations minus incorrect annotations represent total number of the GO terms that the curators should have correctly retrieved from the paper. F-measure = (balanced precision and recall) = 2 × P × R/(P+R).

**Table 6:** Comparison of BioCreAtIvE test set manual annotations with electronic GO annotation predictions.

	**InterPro2GO**	**SPKW2GO**	**EC2GO**	
**Total IEA annotations**	635	385	27	
**Exact term**	151 (0.24)	62 (0.16)	18(0.67)	Correct
**Same lineage > granularity**	24 (0.04)	10 (0.03)	3 (0.11)	Potentially Incorrect/Correct
**Same lineage < granularity**	273 (0.43)	170 (0.44)	1 (0.04)	Correct
**Total same lineage**	297 (0.47)	180 (0.47)	4 (0.15)	Potentially Incorrect/Correct
**New lineage**	187 (0.29)	143 (0.37)	5 (0.19)	Potentially Incorrect/Correct
**Total potential incorrect**	211 (0.33)	153 (0.40)	8 (0.30)	
**Total minimal correct**	424 (0.67)	232 (0.60)	19 (0.70)	
**Precision**	0.67–1.00	0.60–1.00	0.70–1.00	

Where the GO evidence code IEA is 'Inferred from Electronic Annotation' [27]. 'Same lineage > granularity' means where the electronic mapping (InterPro2GO, EC2GO or SPKW2GO) predicted a GO term that was in the same lineage/branch as the manually curated GO term but represented a more granular/parent term. 'Total potential incorrect' annotations = 'Same lineage >granularity' + 'New lineage'. 'Total minimal correct' annotations = 'Exact term' + 'Same lineage < granularity'. Percentage calculations are represented in parentheses.

**Table 7:** Manual verification of electronic GO annotation reliability on 44 proteins.

	**InterPro2GO**	**SPKW2GO**	**EC2GO**	**Grand total**
**Total Proteins**	44	44	44	44
**Total Proteins **(+ predictions)	29 (0.65)	25 (0.56)	11 (0.25)	-
**Total Proteins **(- predictions)	15 (0.34)	19 (0.43)	33 (0.75)	-
**No. GO terms predictions**	107 (0.63)	53 (0.30)	11 (0.06)	171
**Correct**	97	48	11	156
**Incorrect**	0	1	0	1
**Unknown**	10	4	0	14
**Exact term**	40	20	10	70
**Same lineage**	57	28	1	86
**New lineage**	10	5	0	15
**Precision**	0.91–1.00	0.91–0.98	1.00	0.91–0.99

Percentage calculations are represented in parentheses.
