# Supplementary figures and images for: An evaluation of GO annotation retrieval for BioCreAtIvE and GOA
Source: BMC Bioinformatics. 2005 May 24;6(Suppl 1):S17. doi: 10.1186/1471-2105-6-S1-S17 (PMC1869009; doi:10.1186/1471-2105-6-S1-S17)

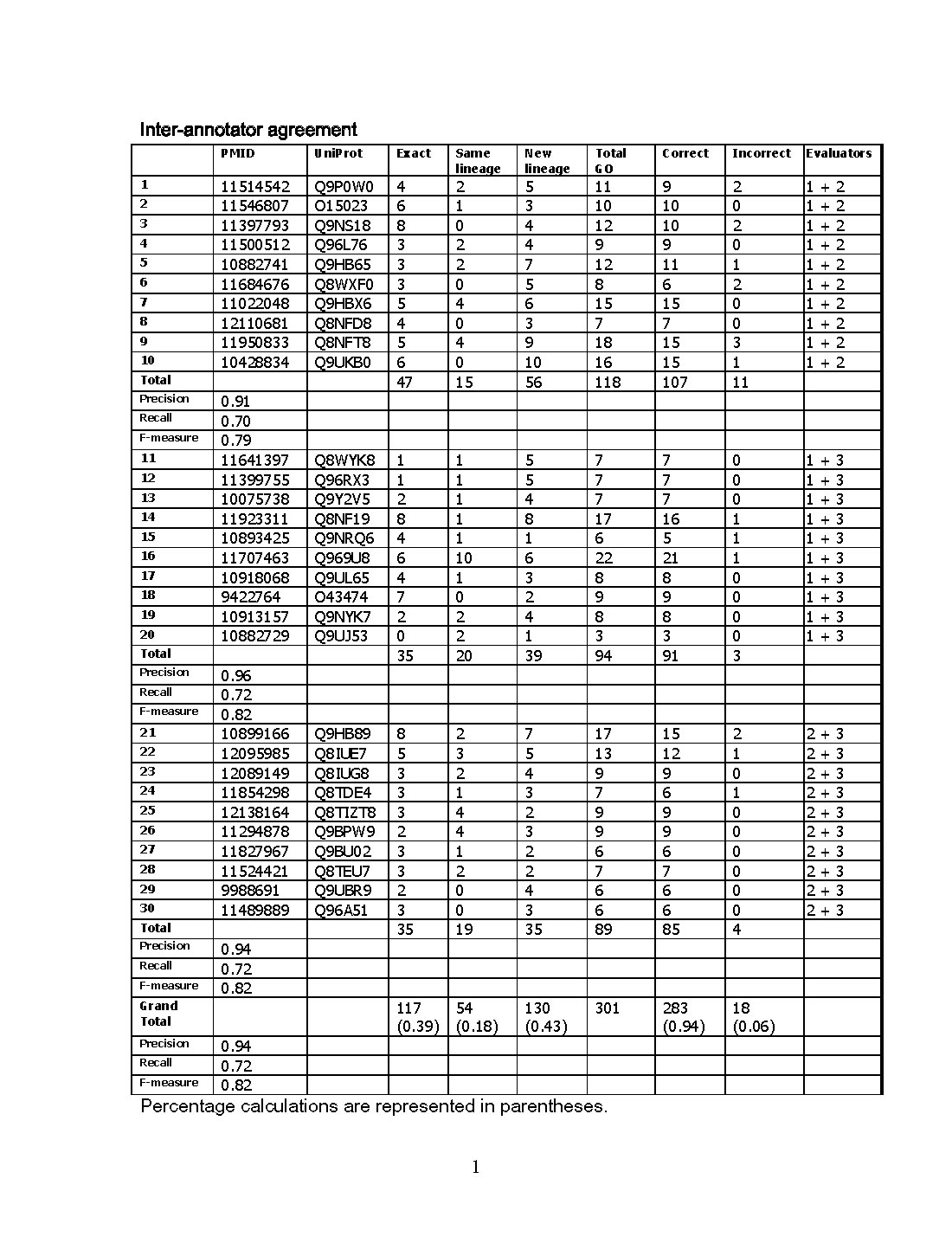

Supplement: Additional File 1 — This shows further details of the inter-annotator agreement. It contains individual counts for each UniProt accession and PubMed Identifier that was co-curated. [file 1471-2105-6-S1-S17-S1.jpg]
